# Understanding interactive effects between habitat configuration and pesticide use for pollination: towards better informed landscape management

**DOI:** 10.1186/s13717-025-00587-z

**Published:** 2025-03-03

**Authors:** Swantje Gebhardt, Jerry van Dijk, Marjolein E. Lof, Martin J. Wassen, Martha Bakker

**Affiliations:** 1 Environmental Sciences, Copernicus Institute of Sustainable Development, Utrecht University, Princetonlaan 8a, PO Box 80115, 3508 TC Utrecht, the Netherlands; 2 Landscape Architecture and Spatial Planning Group, Department of Environmental Sciences, Wageningen University & Research, Gebouw 101 ESG/LSP, Droevendaalsesteeg 3, 6708 PB Wageningen, the Netherlands; 3 Earth Systems and Global Change Group, Wageningen University & Research, Droevendaalsesteeg 3a, 6708 PB Wageningen, The Netherlands

**Keywords:** Land use configuration, Pollination service, Pesticide exposure, Habitat restoration, Simulated landscape

## Abstract

**Background:**

The restoration of natural landscape elements is a frequently adopted pathway to improve wild pollinator abundance, diversity, and their pollination services in intensively used agricultural landscapes. However, pollinators in the intended refuges can become exposed to agrochemicals when foraging in surrounding agricultural fields. In order to effectively design pollinator conservation measures such as habitat restoration or pesticide reduction schemes, the effect of land use configuration on pesticide exposure and pollination service requires further investigation.

**Methods:**

We developed a pollination model that extends existing approaches by simulating both pollination flights and concurrent pollinator exposure to toxic pesticides, enabling the estimation of pesticide impacts on pollination services. We calculated pollination service and pollinator health for a set of artificial landscapes, which varied in the percentage of pollinator habitat and agriculture, in the clustering of these land uses, as well as in the pollinator mortality hazard arising from the pesticides applied on agriculture.

**Results:**

Our results show that in landscapes with less than 10% habitat and highly toxic pesticides, pollination services are mostly safeguarded by compact patches of habitat, as this configuration shelters more habitat from pesticide exposure. With increasing habitat amount or with pesticide applications causing less than 50% mortality in pollinators, more dispersed patches of habitat achieve a better pollination service for the landscape. We further tested the effect of pesticide application for different foraging ranges in a more realistic land use scenario. For pollinators with shorter foraging ranges, pesticide exposure from the immediate surroundings determines the achieved pollination. For species with longer foraging ranges, the availability of resources and the application of pesticides at landscape scale controls the pollination.

**Conclusion:**

Our study highlights the importance of assessing spatial configuration effects on pesticide exposure for local pollinators. By applying these insights, land managers can devise land use arrangements to protect pollinator habitats and establish buffer zones to support pollinator activity in pesticide-intensive landscapes. As current guidelines largely lack spatially-explicit measures, we suggest to direct future research and policies towards the underlying spatial processes and their facilitation on parcel, farm, and landscape scale.

**Supplementary Information:**

The online version contains supplementary material available at 10.1186/s13717-025-00587-z.

## Background

Animal pollination is a crucial ecological process, essential for the functioning of natural ecosystems and the production of over 75% of globally significant food crops (Klein et al. 2007; IPBES 2016). Pollination from wild bees plays a particularly vital role in enhancing the growth and quality of crops such apples, pears, rapeseed, various berries, and cucumbers (Klatt et al. 2014). However, the conditions for wild pollinators in agricultural landscapes have been deteriorating due to the removal of field borders, hedgerows, fallows, and tree groves (Hass et al. 2018; Martin et al. 2019). Furthermore, pollinator health and activity suffer the effects of pesticides applied on agricultural fields (Mancini et al. 2019; Main et al. 2020). In interaction with climate change and parasite pressure, these intensive agricultural practices have led to a decline in pollinator diversity and abundance. Globally, the number of recorded bee species dropped by approximately 25% between 2006 and 2015 compared to pre-1990 levels (Zattara and Aizen 2021). While regional trends in pollinator abundance vary, declines in managed honeybees and wild bees are particularly well-documented in highly industrialized regions such as North America and Western Europe (Goulson 2013; IPBES 2016; Mancini et al. 2019).

To increase pollination service from wild pollinators, many studies suggest that higher percentages of natural and semi-natural land uses could provide resources and refuge to pollinators (Park et al. 2015; Nicholson et al. 2017). The addition of pollinator habitat has been shown to be particularly effective in intensively managed agricultural landscapes with high use of agrochemicals (Carrié et al. 2017; Marja et al. 2019). Hence, increasing the amount of natural landscape elements in agricultural landscapes is a frequently recommended measure to halt pollinator decline (Mottershead and Underwood 2021). For instance, the EU Farm to Fork and Biodiversity Strategy aims to restore high-diversity landscape features on at least 10% of the agricultural area by 2030. Furthermore, these policies seek to decrease use of harmful pesticides by 50% (European Commission 2020). This set of goals is complemented by the revised EU Pollinator Initiative, which lists more detailed objectives to establish knowledge on pollinator decline, increase habitat restoration, and facilitate cooperation among stakeholders on multiple levels (European Commission 2023). Although these policies pursue an integrated approach, a major shortcoming is their lack of spatially-explicit and landscape-oriented strategies for pollinator conservation in areas of intensive agricultural land use. The current policies do not account for the interactive effects of pollinator habitats and pesticide application in given spatial arrangements, even though they are a key driver of pesticide risk for pollinators (Nicholson et al. 2024). During their foraging activities, pollinators come into contact with or ingest contaminated nectar and pollen, especially in close-by, bee-attractive crops (Lonsdorf et al. 2024). Higher exposure leads to declines in pollinator population and activity, consequently reducing the pollination service provided by the affected bees (Van Den Brink et al. 2021). Therefore, the positive effects of semi-natural elements are limited when pollinators in the intended refuges become exposed to pesticides applied in their surroundings (Kohler et al. 2008; Bloom et al. 2021). In sum, the landscape-scale configuration of land uses determines the effectiveness of pollinator habitat restoration or pesticide use reduction on specific fields, and should therefore be included in guidelines for pollinator conservation and pollination management.

The risk of pesticides for pollinators has to be better understood in different landscape contexts to inform policies regarding the benefit of, for instance, flower plantings, or pertaining to the risk for pollinator species with different behavior (Rundlöf et al. 2022; Knapp et al. 2023). The effect of pesticides on pollinator communities is examined in various environmental risk assessments and pollinator colony models (Schmolke et al. 2019; Faber et al. 2021; European Food Safety Authority 2023), but it is, to our knowledge, not yet included in any approach to model pollination services. Thereby, it has not been clarified how the spatial arrangement of agriculture with pesticide use and pollinator habitat patches affects pollination service.

To address this knowledge gap, we developed a model that simulates the spatial processes of pollinator exposure to toxic pesticides and the subsequently reduced pollination service. We first use the model on artificial landscapes to better understand the effects of spatial configuration in interaction with pesticide use as a driver of pollination. With a systematic approach, we explore the average pollination service and pollinator health in landscapes that vary in terms of percentage of pollinator habitat, pesticide hazard on agriculture, and spatial clustering of the two land cover types. Secondly, we use the model to analyze how the placement of pollinator habitat affects pollination service at pollination-dependent crop fields under pesticide exposure in a more realistic land use scenario. Our goal is to identify which specific land use configurations are most effective for supporting pollination services and conserving a variety of pollinator species in areas that are intensively managed for agricultural production. From our results, we discuss how strategic spatial management of land use could enhance the success of pollinator-friendly strategies, such as habitat restoration and the reduction of pesticide use. Finally, we outline opportunities for future research, particularly focusing on the inclusion of spatially-explicit pesticide exposure in pollination service and the effect of other land uses on pollination.

## Methods

### Creating land use composition and configuration gradients

To investigate the interactions of land use composition and configuration with pesticides use on agricultural land, we simulated a large set of binary landscapes covering the gradients of habitat percentage and land use clustering. Using Neutral Landscape Modelling in R (Sciaini et al. 2018; R Core Team 2024), we created random patterns of agricultural and habitat land use clusters. To define intervals within the gradient of habitat percentage, we referred to the available potential pollinator habitat, such as hedgerows and tree lines on agricultural land. As the average percentage of these habitats ranges from 2.6% to 9.3% in EU countries (Czúcz et al. 2022), we included more intervals at the lower end of the habitat percentage gradient, resulting in the following steps: 3%, 5%, 10%, 15%, 30%, and 50%.

**Fig. 1:**
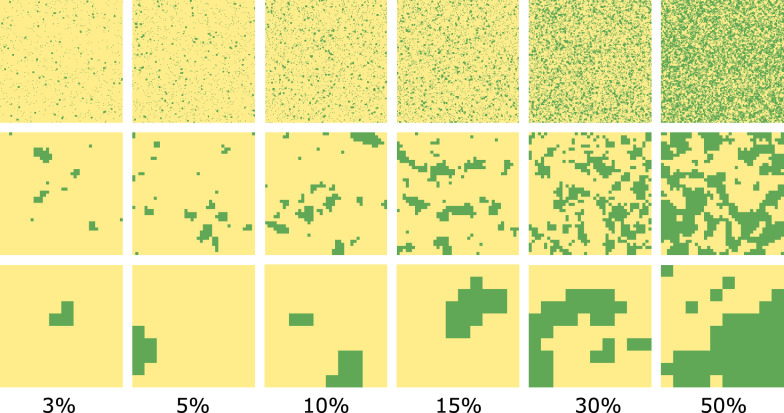
Examples of simulated landscapes with habitat (green) and agriculture (yelllow) at three levels of spatial clustering for each habitat percentage interval

To describe the variation of landscape configuration relevant for the processes we simulate in our pollination model, we created a gradient of land use clustering levels. We first generated landscapes with different cell sizes, each containing random patterns with varying degrees of clustering between the two land uses. After resampling the landscapes to the same cell size, we calculated the log-transformed landscape shape index for each landscape. This metric describes the dispersion of land use classes through measuring the total edge length between these classes standardized by the size of the landscape (McGarigal 2015). Using this index, we arranged the created landscapes along a gradient from dispersed to clustered land uses. Finally, we selected those landscape settings (cell size and degree of land use clustering) that represented approximately equal intervals along our clustering gradient. Corresponding to every interval step of the habitat percentage gradient, a gradient of clustering was produced with the selected landscape settings, ranging from finely interspersed patterns to large and compact blocks of habitat and agriculture (Fig. 1). We ran 50 repititions of each landscape setting to account for variations in the land use composition and clustering.

In addition to a variety of social behaviors, different species of pollinators can have widely varying foraging distances. While small sweat bees forage within a 200 m radius, medium-sized mason bees fly between 400 and 1000 m, and bumble bees and honey bees can cover large distances between 1500 and 3000 m (Hladik et al. 2016; Knapp et al. 2023; Pashanejad et al. 2023). We therefore modelled pollination for several foraging ranges in a preliminary analysis, revealing that landscape dimensions, cell size, and foraging range determined the effect of the land use clustering level on pollination (see Supplementary Material). Consequently, only one foraging range was necessary in combination with the appropriate landscape dimensions and cell size to model the interaction of land use configuration and pesticide use. We chose to set the dimensions of our landscapes to 2000 × 2000 m, the cell size to 10 m, and the foraging radius to 500 m to accommodate both a large gradient of clustering and to limit computation time.

### Including pesticide exposure in the pollination model

Pollinators are exposed to pesticides via dermal contact, oral intake, or inhalation during their pollination flights. The ecotoxic effect of pesticide substances on pollinators depends on a range of interacting factors such as the pollinators’ foraging behavior, body size, and consumption mass, volume, timing and method of pesticide application, as well as the toxicity of the applied substance (Crenna et al. 2020; European Food Safety Authority 2023). As our study investigates the interactions between habitat arrangement in landscapes and pesticide effect, we refrained from calculating pesticide-induced mortality for specific crop treatment cases. Instead, we simulated pollinator health and pollination along a theoretical gradient of pollinator mortality hazard from pesticides use, hereafter called hazard. We use this theoretical hazard gradient in place of measured toxic loads from pesticide application mass and toxicity (Douglas et al. 2020). We ran our model over the simulated landscapes for five hazard levels assigned to the agricultural land use: 0%, 25%, 50%, 75%, 100%. These levels are related to the classification of unwanted lethal pesticide side effects that are published for specific dosages of available pesticide products (IOBC 2022). As these side effect classes are characterized as mortality rates for a group of mixed species (Biobest Group 2024), we utilized this classification to appoint the levels of our hazard gradient.

In the first part of our model, the pollinator flights and the subsequent exposure to pesticide hazard are simulated with a modelling approach presented by Lonsdorf et al. (2024). Using the published R code for their “Spatially Explicit Model of Landscape Exposure (SEMLE)” (Zenono 2023), we first calculate the exposure of pollinators at a given site through simulating their foraging behavior. Through a kernel smoothing calculation, the hazard within the foraging range is summarized based on the probability of a pollinator visiting a location, which exhibits a distance-decay function. While the original model in Lonsdorf et al. (2024) simulates landscape pesticide exposure as described, we treat the calculated numbers as the incurred mortality hazard. As such, the resulting values are substracted from the initial pollinator capacity (100%) in the respective habitat raster cell. The result is the remaining pollinator capacity, which we refer to as pollinator health in our results to reflect how much damages the pesticide exposure has caused. In our model, we further calculate pollination service as the visitation of pollinators to the agricultural fields. For this, our model utilizes again a kernel-smoothing approach to estimate the probability of pollinators arriving at an agricultural field location based on the reduced capacity or pollinator health in the habitats.

### Land use scenario design

In addition to examining the effects of pesticide exposure across varying levels of land use clustering and different percentages of habitat, we also investigated how the placement of habitat areas and agricultural fields influenced pollination for pollinators with different foraging ranges. Specifically, we assessed how the distance between areas where pollinators are nesting and areas where crops require pollination affects pollination services under high pesticide pressure. To better understand how pollination service is vulnerable to pesticide effects, we designed a landscape with different crop types, of which one needs pollination and simultaneously has pesticides applied on it with 75% hazard. The land use scenario also features four small habitat areas, two of which are in direct neighborhood to the agricultural fields with pesticide application, and two which are in between fields with no pesticide use and no pollination demand. This allocation choice allowed us to compare the pollination service and pollinator health in this land use scenario for two pollinator species with different foraging ranges.

**Fig. 2:**
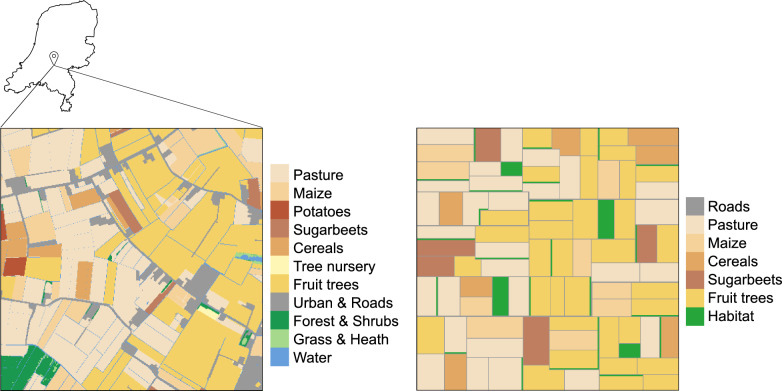
Land use in the Batavia region (left) used as basis for our land use scenario design (right)

We based the scenario on the land use maps (Hazeu et al. 2023) in the Batavia region (Betuwe, in Dutch) in the Netherlands (Fig. 2). In this area, wild pollination is highly relevant for yield and quality of apples, pears, and berries (Reemer and Kleijn 2012; de Groot et al. 2015). Conversely, these fruit trees are treated throughout the year with high amounts of pesticides compared to other crops, including with substances that are harmful to pollinators (CBS 2020; PPDB 2024). Further, the landscape in this region is characterized by large rectangular fields with few hedgerows and small percentage of natural land uses on agricultural land (Hazeu et al. 2023). Finally, the Netherlands are facing a strong decline in pollinator diversity (LVVN 2023), making this case specifically suited for testing the effects of land use arrangement on pollination service and pollinator conservation.

In the fruit orchards of the Batavia region, the most prominent wild bee pollinators are *Andrena* and *Bombus* species including *Bombus terrestris*, *Bombus lapidaries*, *Bombus pascuorum*, *Andrena haemorrhoa*, *Andrena flavipes*, *Andrena cineraria*, and *Andrena nitida* (Reemer and Kleijn 2012; Hutchinson et al. 2021). As the foraging ranges of those pollinator species can differ substantially (Zurbuchen et al. 2010), we decided to calculate and compare the pollination service and pollinator health in our land use scenario for foraging ranges of 400 m and 1000 m.

## Results

### Average pollination service and pollinator health in artificial landscapes

**Fig. 3:**
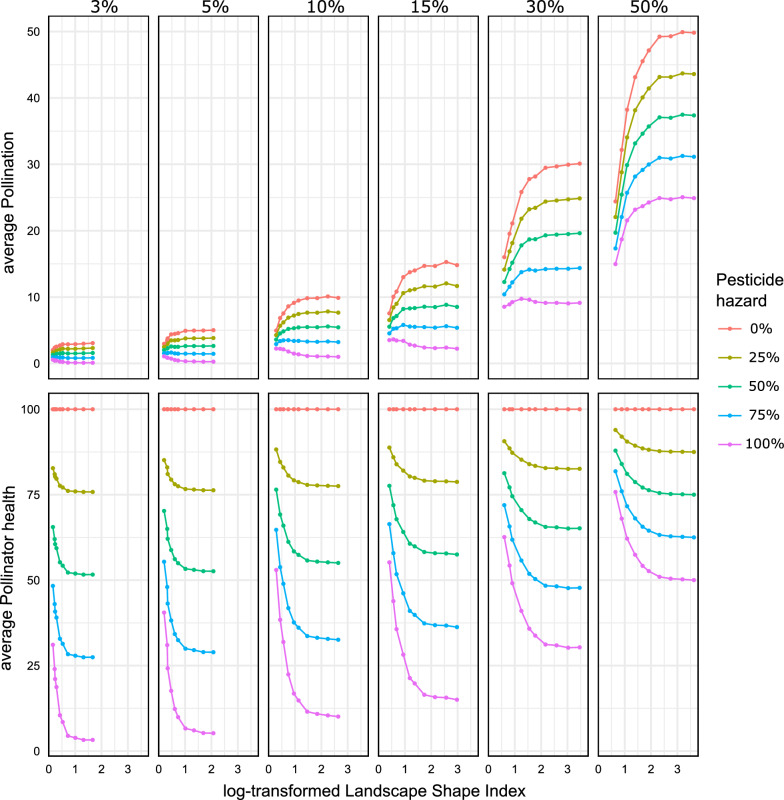
Average pollination service on agriculture (above) and pollinator health in habitats (below) across the gradient of habitat percentage (panels), pesticide hazard level on agricultural land (colored lines), and land use clustering (*x*-axis). Lower values of the log-transformed landscape shape index, log(LSI), refer to more clustered land uses and high values refer to more interspersed land uses

The average pollination service and pollinator health varied along the gradients of habitat percentage, land use clustering, and pesticide hazard level (Fig. 3). As expected, both pollination service and pollinator health increased with habitat percentage, as each habitat cell was set as a source of pollinators with an initial pollination capacity of 100%. Furthermore, both result variables decreased with increasing pesticide hazard across all levels of habitat percentage and land use clustering.

Pollination service varied strongly between the hazard levels in landscapes with an equal distribution of habitat and agricultural land use (panel on the right-most position, Fig. 3). Differences in pollination service between hazard levels within the same level of habitat percentage were highest when the land uses were more dispersed (within panels at high log(LSI) values, Fig. 3). In both cases, natural habitat and agriculture had a higher probability of being interspersed. In turn, the amount of habitat area that was within short distance from agriculture with pesticide use increased and subsequently the pesticide hazard level had greater influence on the resulting pollination service in the landscape.

With increasing habitat percentage, pollination service improved gradually across all hazard levels in landscapes where habitat was more interspersed with agriculture. Along the gradient of habitat percentage, pollination service values also varied according to the interaction of land use configuration and pesticide hazard. In landscapes with a small habitat percentage, pollination service increased with stronger land use clustering if the hazard level was high. In contrast, if the hazard was lower, pollination increased with more dispersed land use classes. At first, landscapes of higher hazard showed constant pollination service, but from approximately 30% habitat onward, pollination services in these landscapes also surged strongly in landscapes with little clustering.

The variation in pollinator health between hazard levels was highest in landscapes with small habitat percentage and interspersed land uses. With little habitat available, the impact of hazard levels increased as these landscapes were largely dominated by agriculture from which the pesticide residues were collected. Landscapes with more clustered land uses performed better in terms of pollinator health across the gradients of habitat percentage and hazard level, as they host more compact habitat patches. In bigger and spatially condensed habitat areas, pollinators foraging from the centers of these patches were less exposed to pesticides as their foraging area included smaller shares of agriculture.

### Effect of foraging range and spatial context of habitat and agriculture

**Fig. 4:**
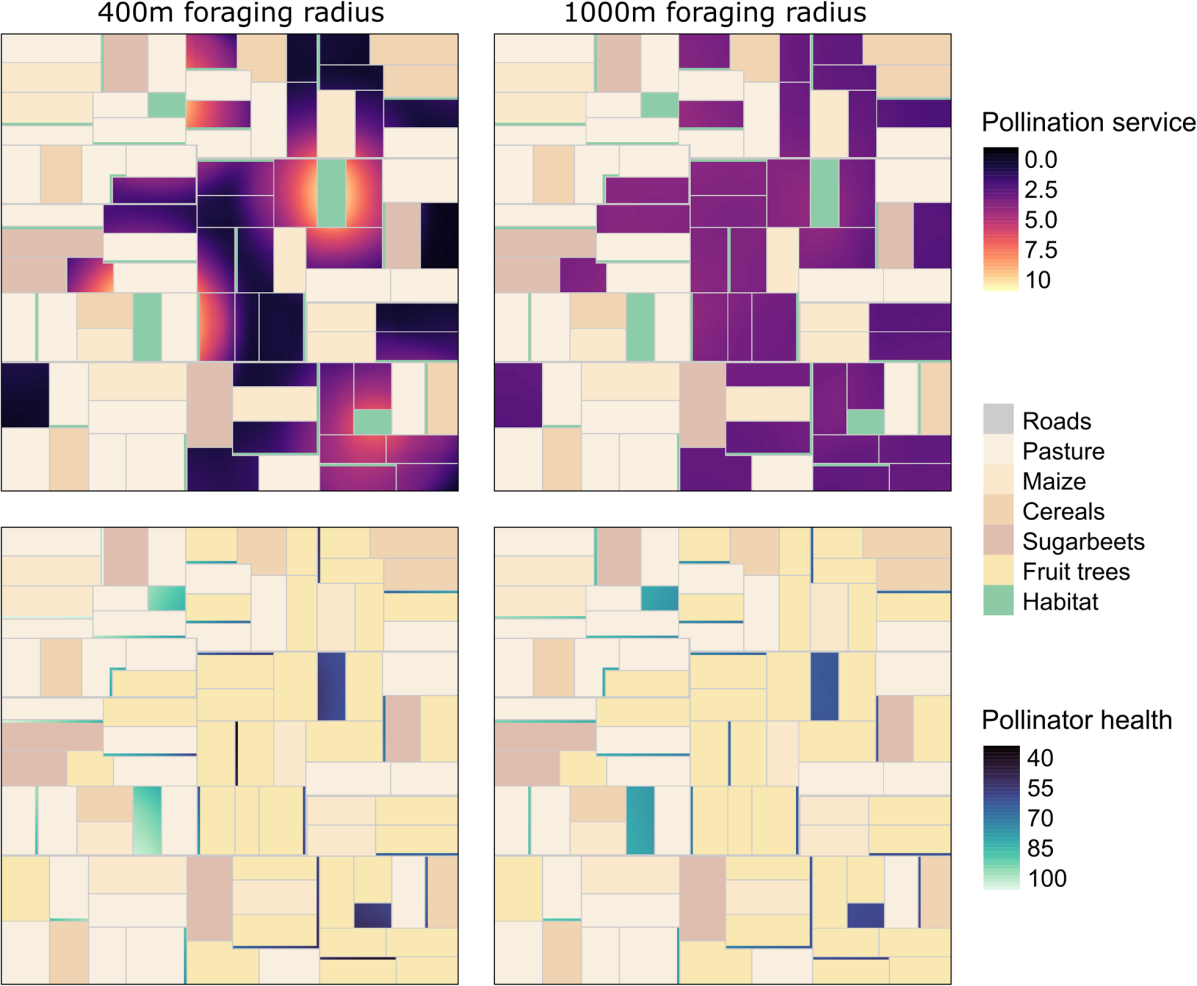
Spatial visualization of modelled pollination service on fruit trees (top maps) and pollinator health in natural habitat (bottom maps). Both values are shown over the land use scenario (land use types indicated in the middle legend panel) based on the Batavia region. For clarity, the maps display the actual value range achieved in this scenario, out of the possible range of 0 to 100 for pollination service and pollinator health. Maps on the left represent results for a 400 m foraging range; maps on the right for a 1000 m foraging range

In the land use scenario based on fruit cultivation in the Batavia region in the Netherlands, simulations for pollinators with different foraging ranges resulted in different spatial patterns of pollination services on the fruit tree parcels (Fig. 4). With a 400 m foraging range, hot spots of pollination service were located directly around the habitat patches. With increasing distance from the habitats, higher pollination was only achieved in fruit tree fields that were within the foraging distance of the two habitats that received less pesticide exposure. The more isolated fruit trees received almost no pollination. In our scenario, pollinators with shorter foraging range ecountered almost exclusively agriculture with high pesticide hazard, if their habitats were surrounded by fruit trees. Consequently, pollination was reduced to similar values as the pollination in fruit trees far away from habitat patches. Pollinators with 1000 m foraging range were able to reach large parts of the landscape in our scenario. Pollination service values were almost the same in most fruit tree parcels across the landscape, as the pollinators from each habitat were exposed to similar amounts of pesticide-treated agriculture. This effect was caused by the larger foraging range of the pollinators and the distribution of habitat and agricultural fields with pesticide use in our scenario: pollinators from habitats surrounded by fruit trees also reached fields with no pesticide hazard during their foraging, and pollinators from habitats surrounded by pesticide-free fields also flew into fruit tree fields and were exposed to pesticides there.

Since pollinator health was a reflection of pesticide exposure, the resulting values showed the same patterns as the pollination service. Namely, pollinator health was considerably higher in habitats outside of the fruit tree concentration for short range foragers. For long range foragers, the pollinator health in all habitats was more similar, since the area for potential pesticide exposure was larger and covered also fields with no pesticide use.

## Discussion

### Configuration modified pesticide exposure effects on pollination service and pollinator health

By calculating pollination service and pollinator health across gradients of habitat percentage, land use clustering, and pesticide hazard, our model gave insights into the spatial dynamics of pesticide exposure. Our results demonstrated that land use dispersion tends to enhance pollination service in landscapes with low pesticide hazard. Conversely, in landscapes with high pesticide hazard, land use clustering benefits pollination service and pollinator health. Importantly, our findings show that the combination of habitat percentage and pesticide hazard determines the point at which land use clustering stops being beneficial and land use dispersion becomes more advantageous. Thus, the overall availability of habitat plays a critical role in shaping how land use configuration affects pollination under pesticide pressure. We also demonstrated that the tradeoff between pollinator health and pollination is largely influenced by the pollinator's foraging range, as it defines both the area that can recieve pollination and the area where pollinators are at risk of potential pesticide exposure. By considering the pesticide hazard level, pollination demand, and the species’ foraging range, pollinator habitats can be strategically positioned to try to alleviate this tradeoff. Overall, our study illustrated that the combination of land use arrangement and pesticide management affects pollinators and their contribution to agricultural production. These processes occur at landscape-scale, underlining that current policies aiming to conserve pollinators and stimulate pollination as an ecosystem service, such as the Europen Green Deal, can be much more effective if they address coordination at landscape level in addition to the current thresholds for habitat percentages and pesticide use.

Through the inclusion of pesticide effects, we highlighted that agricultural land use intensity modifies the impact of land use arrangement on pollination, which puts existing research into context. In examining current modeling studies, Rahimi et al. (2021a) investigated the interaction between habitat percentage, fragmentation, and habitat capacity to provide pollination in artificial land use scenarios. Their results demonstrated that high-quality patches were more effective when interspersed in the landscape, whereas low-capacity patches needed to be aggregated to improve pollination. These findings align with our results, as we also observed that consolidated habitat patches safeguarded more area in high-risk landscapes, while dispersed patches were more beneficial under low pesticide hazard. Unlike Rahimi et al. (2021a), who based habitat capacity on nesting and floral resources using the widely-used Lonsdorf approach, our study emphasized that habitat capacity is also highly influenced by surrounding land uses. Similarly, Ziółkowska et al. (2021) showed that field margins were not as beneficial to carabid beetles under high insecticide use in the landscape. Their modelling study further clarified that landscape context largely drives the effectiveness of agri-ecological measures.

There are limited ecological field studies that have measured the interactive effect of land use composition, configuration, and pesticide use for pollinators. The existing field studies have demonstrated that increasing the amount of natural areas positively impacts pollination, particularly around farms that use a lot of pesticides (Park et al. 2015; Carrié et al. 2017; Nicholson et al. 2017). However, the values of natural habitat percentage in the respective studies were already quite high, as they varied for instance between 29 and 86% (Nicholson et al. 2017) or between 5 and 64% (Carrié et al. 2017). If the overall habitat percentage in the landscape is high, even fragmented or dispersed habitat patches become connected and provide more pollination service. This relation between habitat fragmentation and habitat percentage was already reported in knowledge synthesis studies and conceptual frameworks (Mitchell et al. 2015; Martin et al. 2019; Maurer et al. 2020), but our study is among the first to clarify that connected habitats safeguard pollinator health through reduced pesticide exposure. Our results confirm that percentage of habitat is a significant factor, but also emphasize that land use configuration determines pollinator health and pollination service in landscapes with little habitat percentage and high pesticide use. We stress that the addition of natural areas around farms to increase pollination on pesticide-treated crops cannot be supported without reservations. The added areas may act as stepping stones to guide pollinators into the fields, but they cannot be considered refuges that protect sensitive species from pesticide effects. Pollinators foraging from such habitat patches can be overwhelmed by their exposure to agrochemicals, particularly if these patches are small, and interspersed with the agriculture. Several field studies found that flower plantings next to pesticide-treated fields are particularly prone to become routes of pesticide exposure (Botías et al. 2015; Mogren and Lundgren 2016). Considering our findings, we call for field studies to evaluate the effectiveness of agri-environmental management strategies under pesticide exposure. Thereby, their assessments would incorporate a major stressor of pollinator health and characterize land use intensity beyond nitrogen input (Marja et al. 2019).

### Landscape planning for pollinator health and pollination service

Our findings provide a foundation for strategic landscape planning that aims to support both pollinator health and pollination services. First, measures to improve the conditions for pollinators must account for the distinct subsets of the landscapes experienced by species with different foraging ranges (De Palma et al. 2015; Knapp et al. 2023). As shown in our simulation, short range foragers were influenced primarily by the land use right around their habitat. Hence, these pollinators require closely connected nesting and floral resources (Cole et al. 2020). To enable short range foragers to pollinate crops that must receive pesticide application, fields should be buffered with a small border of non-toxic land uses. In contrast, our study demonstrated that long range foragers can encounter more diverse land uses in their much larger home range. Therefore, the ideal habitat arrangement to support these pollinators can be spatially more flexible, but long range foragers need resources across the landscape to ameliorate the pesticide effects that they might become exposed to (Rundlöf et al. 2022). Second, our findings also underpin the importance of reducing the overall pesticide use and establishing large nature areas to protect vulnerable species. Landscape planning measures should not focus solely on species that perform most pollination services, but also support species that are threatened by agrochemicals and require specific habitat configurations (Kleijn et al. 2015). Instead, the complementary use of large high-quality patches and heterogenous patterns can be a promising strategy to enhance both species conservation and facilitate pollinator spillover into agriculture (Senapathi et al. 2017; Grass et al. 2019, 2021). In addition, strategic design of landscapes that support biodiversity should include the creation of spatially well-connected and diverse supply of nesting and floral resources (Hladik et al. 2016; López-Cubillos et al. 2023). However, many national conservation strategies for pollinators still fail to fully integrate habitat complementarity and connectivity (Vasiliev and Greenwood 2020).

While current policies rarely consider the benefit of specific natural habitat configurations, there are significant challenges to implementing pesticide use reduction and an increase in compact habitat areas. For instance, in the Netherlands, expanding existing protected areas often conflicts with competing demands for urbanization and industrial development. Additionally, farmers are resistant to the displacement of their agricultural activities (Tisma and Meijer 2018). In contrast, the pressure on natural areas is particularly intense in Latin America, Africa, and Southeast Asia, where extensive agricultural exports drive land-use change (Cabernard et al. 2024). In these regions, protected areas must first be safeguarded against agricultural expansion through robust environmental law enforcement before they can be expanded (Gopel et al. 2020). The reduction of pesticide use also faces numerous barriers. A case study on pome and stone fruit production revealed that farmers view transitioning to alternative pest control methods as risky and fear reduced market acceptance for crops that are not visually perfect (Bravin et al. 2022). Furthermore, small-scale farmers often lack access to the technology and support needed to reduce agrochemical inputs (Dhillon and Moncur 2023). Finally, integrated pest and pollination management requires comprehensive knowledge of biotic and abiotic processes, as well as of the impacts of field and landscape management practices (Lundin et al. 2021). With our study, we address the latter challenge by illustrating how the spatial context of land use influences the benefits of natural elements.

### Pollination model expansion and further research

To systematically explore the interactive effects of land use arrangement and pesticide hazard on pollinator health and their pollination services, we applied our pollination model on artificial landscapes with uniform land use properties. This proof-of-principle approach enabled us to isolate and examine the individual effects of each factor, providing a clearer understanding of the underlying processes and interactions. Consequently, while our findings provide insights into the spatial mechanisms, they cannot be directly applied to real landscapes due to the highly variable effects of pesticide exposure on pollination services in heterogeneous land use patterns. First, predicting pollination in real landscapes requires replacing the homogeneous pesticide hazard assigned to agriculture in our simulated landscapes with data on actual pesticide application. Local mortality risk can be estimated using various approaches, such as combining pesticide load and toxicity data for agrochemicals applied to different crops (Douglas et al. 2022). Second, modeling pollination in more realistic landscapes should account for habitats with varying carrying capacities for pollinators, as these differences influence the local potential for pollination (Lonsdorf et al. 2009; Rahimi et al. 2021b). Third, real landscapes add complexity because different crop types have varying pollination demands (Klein et al. 2007). Lastly, land uses between supply and demand areas can play a significant role for the ecosystem service flow (Assis et al. 2023). For example, roads are fragmenting nature and can cause roadkill in pollinators (Olynyk et al. 2021), while urban green spaces can supply additional floral resources (Theodorou et al. 2020).

Beyond changes in the representation of land uses, pollination models could incorporate more specific pollinator behavior, such as preferential foraging flights to floral resources of high quality, to emphasize the impact of spatial configuration. Specifically in heterogeneous landscapes, the inclusion of a behavioral component could result in different results for pollination and pesticide exposure as the pollinators experience a different section of the landscape compared to a purely distance-based flight simulation (Olsson et al. 2015; Knapp et al. 2023). In future applications, pollination models could also include the impact of agrochemicals that indirectly affect pollinators. Fertilizer and herbicide application alter plant growth and flowering characteristics of pollinator floral resources (Banaszak-Cibicka et al. 2019; Carpenter et al. 2020; Belsky and Joshi 2020). As our modelling approach focussed on the landscape-scale, we did not account for pesticide drift. However, we recommend to incorporate this factor for field- or farm-scale studies, as pesticides commonly drift onto field margins (Holterman and van de Zande 2021). These vegetation strips can receive between 5 to 25% of the field application rate, which can impact their floral resources and hence alter the pollination services in the surroundings (Dupont et al. 2018).

As our study was informed by both ecological risk assessment and ecosystem service research, we also recommend further work to bridge the gap between these fields. While decision-support tools exist that specifically target pollination service, e.g. Beescape (Robinson et al. 2021), ecosystem service mapping applications (e.g. InVEST, ARIES, ESTIMAP) could benefit from incorporating the spatially-explicit pesticide exposure impacts investigated in this study. The pesticide effects could also be included in process-based models to refine the outcomes of landscape optimization studies, such as in Knight et al. (2024). A deeper understanding of pollinator exposure to toxic agrochemicals would provide a more realistic picture of the land use management scenarios analyzed with these tools. Further, decision-support tools that map spatially-explicit ecological processes for users with diverse knowledge backgrounds (Gebhardt et al. 2024) could inform the public on the spatial component of pollinator decline and possibly improve their users’ willingness to support policies for pesticide reductions.

## Conclusion

Through a systematic exploration of pesticide exposure in landscapes across gradients of habitat percentage, land use arrangement, and pesticide hazard, our study adds to the understanding of spatial configuration effects on pollination. Based on our simple modelling framework, our study highlighted the interactive effects of habitat addition, land use configuration, and pesticide use on agriculture on pollinator health and pollination services in landscapes. Particularly, the pollinator exposure to pesticides depended on the size and distribution of habitat patches relative to agricultural fields. In agricultural landscapes with limited amounts of natural landscape features, the findings of our study suggest to invest in more compact habitat patches. Further, the reduction of pesticide use could foster pollination services if applied in strategic locations, such as buffer areas around pollinator habitats to protect pollinators from exposure. Additionally, the foraging range of relevant pollinators should be incorporated in planning pollinator-friendly measures to provide them with resources at reachable distances and enable spillover into pollination-dependent fields.

## Supplementary Information

Supplementary material 1.

## Data Availability

Data will be made available from the corresponding author upon reasonable request. The Model Script “Pollination model with pesticide effects” is available at: https://github.com/s-gebhardt/PollPestEffect

## References

[CR1] Assis JC, Hohlenwerger C, Metzger JP et al (2023) Linking landscape structure and ecosystem service flow. Ecosyst Serv 62:101535. 10.1016/J.ECOSER.2023.101535

[CR2] Banaszak-Cibicka W, Takacs V, Kesy M et al (2019) Manure application improves both bumblebee flower visitation and crop yield in intensive farmland. Basic Appl Ecol 36:26–33. 10.1016/J.BAAE.2019.03.005

[CR3] Belsky J, Joshi NK (2020) Effects of fungicide and herbicide chemical exposure on apis and non-apis bees in agricultural landscape. Front Environ Sci 8:81. 10.3389/FENVS.2020.00081/BIBTEX

[CR4] Biobest Group (2024) Side Effects Data. https://www.biobestgroup.com/side-effects-data. Accessed 05 June 2024

[CR5] Bloom EH, Wood TJ, Hung KLJ et al (2021) Synergism between local- and landscape-level pesticides reduces wild bee floral visitation in pollinator-dependent crops. J Appl Ecol 58:1187–1198. 10.1111/1365-2664.13871

[CR6] Botías C, David A, Horwood J et al (2015) Neonicotinoid residues in wildflowers, a potential route of chronic exposure for bees. Environ Sci Technol 49:12731–12740. 10.1021/ACS.EST.5B03459 26439915 10.1021/acs.est.5b03459

[CR7] Bravin E, Hazic V, Schweiger P (2022) Sustainable ways to reduce pesticides in pome and stone fruit production. Improve farmers position with reduced pesticide use. European Innovation Partnership for Agricultural Productivity and Sustainability (EIP-AGRI)

[CR8] Cabernard L, Pfister S (2024) Hellweg S (2024) Biodiversity impacts of recent land-use change driven by increases in agri-food imports. Nat Sustain 7:1512–1524. 10.1038/s41893-024-01433-4

[CR9] Carpenter DJ, Mathiassen SK, Boutin C et al (2020) Effects of herbicides on flowering. Environ Toxicol Chem 39:1244–1256. 10.1002/ETC.4712 32170767 10.1002/etc.4712

[CR10] Carrié R, Andrieu E, Ouin A, Steffan-Dewenter I (2017) Interactive effects of landscape-wide intensity of farming practices and landscape complexity on wild bee diversity. Landsc Ecol 32:1631–1642. 10.1007/S10980-017-0530-Y/FIGURES/5

[CR11] Centraal Bureau Statistiek (CBS) (2020) Gewasbeschermingsmiddelen in de landbouw; werkzame stof, gewas, toepassing. https://opendata.cbs.nl/statline/#/CBS/nl/dataset/85130NED/table?ts=1718191716972. Accessed 26 May 2024

[CR12] Cole LJ, Kleijn D, Dicks LV et al (2020) A critical analysis of the potential for EU common agricultural policy measures to support wild pollinators on farmland. J Appl Ecol 57:681–694. 10.1111/1365-2664.13572 32362684 10.1111/1365-2664.13572 PMC7188321

[CR13] Crenna E, Jolliet O, Collina E et al (2020) Characterizing honey bee exposure and effects from pesticides for chemical prioritization and life cycle assessment. Environ Int 138:105642. 10.1016/J.ENVINT.2020.105642 32179322 10.1016/j.envint.2020.105642

[CR14] Czúcz B, Bettina B, Terres JM et al (2022) Classification and quantification of landscape features in agricultural land across the EU: a brief review of existing definitions, typologies, and data sources for quantification. Publications Office of the European Union, Luxembourg

[CR15] De Palma A, Kuhlmann M, Roberts SPM et al (2015) Ecological traits affect the sensitivity of bees to land-use pressures in European agricultural landscapes. J Appl Ecol 52:1567–1577. 10.1111/1365-2664.12524 27546902 10.1111/1365-2664.12524 PMC4973690

[CR16] Dhillon R, Moncur Q (2023) Small-scale farming: a review of challenges and potential opportunities offered by technological advancements. Sustainability 15:15478. 10.3390/SU152115478

[CR17] Douglas MR, Sponsler DB, Lonsdorf V, Grozinger CM (2020) County-level analysis reveals a rapidly shifting landscape of insecticide hazard to honey bees (*Apis mellifera*) on US farmland. Sci Rep 10:797. 10.1038/s41598-019-57225-w 31964921 10.1038/s41598-019-57225-w PMC6972851

[CR18] Douglas MR, Baisley P, Soba S et al (2022) Putting pesticides on the map for pollinator research and conservation. Sci Data 9:571. 10.1038/s41597-022-01584-z 10.1038/s41597-022-01584-z PMC9481633 36114185

[CR19] Dupont YL, Strandberg B, Damgaard C (2018) Effects of herbicide and nitrogen fertilizer on non-target plant reproduction and indirect effects on pollination in *Tanacetum vulgare* (Asteraceae). Agric Ecosyst Environ 262:76–82. 10.1016/J.AGEE.2018.04.014

[CR20] European Commission (2020) A Farm to Fork Strategy for a fair, healthy and environmentally-friendly food system (COM/2020/381 final). Available at: https://ec.europa.eu/food/farm2fork_en. Accessed 30 Sep 2024

[CR21] European Commission (2023) Revision of the EU Pollinators Initiative: A new deal for pollinators (COM/2023/35 final). Available at: https://environment.ec.europa.eu/publications/revision-eu-pollinators-initiative-new-deal-pollinators_en. Accessed 12 Oct 2024

[CR22] European Food Safety Authority (2023) Revised guidance on the risk assessment of plant protection products on bees (*Apis mellifera*, *Bombus* spp. and solitary bees). EFSA J 21:e07989. 10.2903/J.EFSA.2023.7989 37179655 10.2903/j.efsa.2023.7989 PMC10173852

[CR23] Faber JH, Marshall S, Brown AR et al (2021) Identifying ecological production functions for use in ecosystem services-based environmental risk assessment of chemicals. Sci Total Environ 791:146409. 10.1016/J.SCITOTENV.2021.146409 33771395 10.1016/j.scitotenv.2021.146409

[CR24] Gebhardt S, Assis JC, Lacayo-Emery M et al (2024) Supporting stakeholder dialogue on ecosystem service tradeoffs with a simulation tool for land use configuration effects. Environ Model Softw 179:106097. 10.1016/j.envsoft.2024.106097

[CR25] Gopel J, Schungel J, Stuch B, Schaldach R (2020) Assessing the effects of agricultural intensification on natural habitats and biodiversity in Southern Amazonia. PLoS ONE 15:e0225914. 10.1371/JOURNAL.PONE.0225914 33237901 10.1371/journal.pone.0225914 PMC7688104

[CR26] Goulson D (2013) REVIEW: an overview of the environmental risks posed by neonicotinoid insecticides. J Appl Ecol 50:977–987. 10.1111/1365-2664.12111

[CR27] Grass I, Loos J, Baensch S et al (2019) Land-sharing/-sparing connectivity landscapes for ecosystem services and biodiversity conservation. People Nat 1:262–272. 10.1002/PAN3.21/SUPPINFO

[CR28] Grass I, Batáry P, Tscharntke T (2021) Combining land-sparing and land-sharing in European landscapes. Adv Ecol Res 64:1–251

[CR29] de Groot GA, van Kats R, Reemer M, et al (2015) De bijdrage van (wilde) bestuivers aan de opbrengst van appels en blauwe bessen; Kwantificering van ecosysteemdiensten in Nederland. Wageningen

[CR30] Hass AL, Kormann UG, Tscharntke T et al (2018) Landscape configurational heterogeneity by small-scale agriculture, not crop diversity, maintains pollinators and plant reproduction in western Europe. Proc Royal Soc B Biol Sci 285:20172242. 10.1098/RSPB.2017.2242 10.1098/rspb.2017.2242 PMC5829195 29445017

[CR31] Hazeu GW, Thomas D, Vittek M, Staritsky I (2023) Landelijk Grondgebruik Nederland 2022 (LGN2022). Version 1. 4TU.ResearchData. dataset. 10.4121/688363cc-8c79-439f-bb0e-fe5d0deb3161.v1

[CR32] Hladik ML, Vandever M, Smalling KL (2016) Exposure of native bees foraging in an agricultural landscape to current-use pesticides. Sci Total Environ 542:469–477. 10.1016/J.SCITOTENV.2015.10.077 26520270 10.1016/j.scitotenv.2015.10.077

[CR33] Holterman HJ, van de Zande J (2021) WUR drift calculator user manual version. Belonging to Software 2:6

[CR34] Hutchinson LA, Oliver TH, Breeze TD et al (2021) Using ecological and field survey data to establish a national list of the wild bee pollinators of crops. Agric Ecosyst Environ 315:107447. 10.1016/J.AGEE.2021.107447

[CR35] IOBC (2022) IOBC-WPRS (International Organisation for Biological and Integrated Control—West Palaearctic Regional Section). https://iobc-wprs.org/ip-tools/pesticide-side-effect-database/. Accessed 15 June 2024

[CR36] IPBES (2016) Summary for policymakers of the assessment report of the Intergovernmental Science-Policy Platform on Biodiversity and Ecosystem Services on pollinators, pollination and food production. Secretariat of the Intergovernmental Science-Policy Platform on Biodiversity and Ecosystem Services, Bonn

[CR38] Klatt BK, Holzschuh A, Westphal C et al (2014) Bee pollination improves crop quality, shelf life and commercial value. Proc Royal Soc B Biol Sci 281:20132440. 10.1098/RSPB.2013.2440 10.1098/rspb.2013.2440 PMC3866401 24307669

[CR39] Kleijn D, Winfree R, Bartomeus I et al (2015) Delivery of crop pollination services is an insufficient argument for wild pollinator conservation. Nat Commun 6:7414. 10.1038/NCOMMS8414 26079893 10.1038/ncomms8414 PMC4490361

[CR40] Klein AM, Vaissière BE, Cane JH et al (2007) Importance of pollinators in changing landscapes for world crops. Proc Royal Soc B Biol Sci 274:303–313. 10.1098/RSPB.2006.3721 10.1098/rspb.2006.3721 PMC1702377 17164193

[CR41] Knapp JL, Nicholson CC, Jonsson O et al (2023) Ecological traits interact with landscape context to determine bees’ pesticide risk. Nat Ecol Evol 7(4):547–556. 10.1038/s41559-023-01990-5 36849537 10.1038/s41559-023-01990-5 PMC10089916

[CR42] Knight E, Balzter H, Breeze TD et al (2024) Adapting genetic algorithms for multifunctional landscape decisions: a theoretical case study on wild bees and farmers in the UK. Methods Ecol Evol 15:2153–2167. 10.1111/2041-210X.14424

[CR43] Kohler F, Verhulst J, Van Klink R, Kleijn D (2008) At what spatial scale do high-quality habitats enhance the diversity of forbs and pollinators in intensively farmed landscapes? J Appl Ecol 45:753–762. 10.1111/J.1365-2664.2007.01394.X

[CR44] Ministerie van Landbouw, Visserij, Voedselzekerheid en Natuur (LVVN) (2023) 5 jaar Nationale Bijenstrategie 2018–2023

[CR45] Lonsdorf E, Kremen C, Ricketts T et al (2009) Modelling pollination services across agricultural landscapes. Ann Bot 103:1589–1600. 10.1093/AOB/MCP069 19324897 10.1093/aob/mcp069 PMC2701767

[CR46] Lonsdorf EV, Rundlöf M, Nicholson CC, Williams NM (2024) A spatially explicit model of landscape pesticide exposure to bees: development, exploration, and evaluation. Sci Total Environ 908:168146. 10.1016/j.scitotenv.2023.168146 37914120 10.1016/j.scitotenv.2023.168146

[CR47] López-Cubillos S, McDonald-Madden E, Mayfield MM, Runting RK (2023) Optimal restoration for pollination services increases forest cover while doubling agricultural profits. PLoS Biol 21:e3002107. 10.1371/JOURNAL.PBIO.3002107 37220120 10.1371/journal.pbio.3002107 PMC10204975

[CR48] Lundin O, Rundlöf M, Jonsson M et al (2021) Integrated pest and pollinator management—expanding the concept. Front Ecol Environ 19:283–291. 10.1002/FEE.2325

[CR49] Main AR, Hladik ML, Webb EB et al (2020) Beyond neonicotinoids—wild pollinators are exposed to a range of pesticides while foraging in agroecosystems. Sci Total Environ 742:140436. 10.1016/j.scitotenv.2020.140436 32623160 10.1016/j.scitotenv.2020.140436

[CR50] Mancini F, Woodcock BA, Isaac NJB (2019) Agrochemicals in the wild: identifying links between pesticide use and declines of nontarget organisms. Curr Opin Environ Sci Health 11:53–58. 10.1016/j.coesh.2019.07.003

[CR51] Marja R, Kleijn D, Tscharntke T et al (2019) Effectiveness of agri-environmental management on pollinators is moderated more by ecological contrast than by landscape structure or land-use intensity. Ecol Lett 22:1493–1500. 10.1111/ELE.13339 31286628 10.1111/ele.13339

[CR52] Martin EA, Dainese M, Clough Y et al (2019) The interplay of landscape composition and configuration: new pathways to manage functional biodiversity and agroecosystem services across Europe. Ecol Lett 22:1083–1094 30957401 10.1111/ele.13265

[CR53] Maurer C, Bosco L, Klaus E et al (2020) Habitat amount mediates the effect of fragmentation on a pollinator’s reproductive performance, but not on its foraging behaviour. Oecologia 193:523–534. 10.1007/S00442-020-04658-0/FIGURES/4 32333093 10.1007/s00442-020-04658-0

[CR54] McGarigal K (2015) FRAGSTATS Help. http://www.umass.edu/landeco/research/fragstats/documents/fragstats.help.4.2.pdf

[CR55] Mitchell MGE, Suarez-Castro AF, Martinez-Harms M et al (2015) Reframing landscape fragmentation’s effects on ecosystem services. Trends Ecol Evol 30:190–198 25716547 10.1016/j.tree.2015.01.011

[CR56] Mogren CL, Lundgren JG (2016) Neonicotinoid-contaminated pollinator strips adjacent to cropland reduce honey bee nutritional status. Sci Rep 6:29608. 10.1038/srep29608 27412495 10.1038/srep29608 PMC4944152

[CR57] Mottershead D, Underwood E (2021) Pollinators in the CAP: integrating pollinator conservation into the Common Agricultural Policy. Brussels

[CR58] Nicholson CC, Koh I, Richardson LL et al (2017) Farm and landscape factors interact to affect the supply of pollination services. Agric Ecosyst Environ 250:113–122. 10.1016/j.agee.2017.08.030

[CR59] Nicholson CC, Lonsdorf EV, Andersson GKS et al (2024) Landscapes of risk: a comparative analysis of landscape metrics for the ecotoxicological assessment of pesticide risk to bees. J Appl Ecol 61:975–986. 10.1111/1365-2664.14622

[CR60] Olsson O, Bolin A, Smith HG, Lonsdorf EV (2015) Modeling pollinating bee visitation rates in heterogeneous landscapes from foraging theory. Ecol Model 316:133–143. 10.1016/J.ECOLMODEL.2015.08.009

[CR61] Olynyk M, Westwood AR, Koper N (2021) Effects of natural habitat loss and edge effects on wild bees and pollination services in remnant prairies. Environ Entomol 50:732–743 33492391 10.1093/ee/nvaa186

[CR62] Park MG, Blitzer EJ, Gibbs J et al (2015) Negative effects of pesticides on wild bee communities can be buffered by landscape context. Proc Royal Soc B Biol Sci 282:20150299. 10.1098/RSPB.2015.0299 10.1098/rspb.2015.0299 PMC4590442 26041355

[CR63] Pashanejad E, Thierry H, Robinson BE, Parrott L (2023) The application of semantic modelling to map pollination service provisioning at large landscape scales. Ecol Model 484:110452. 10.1016/J.ECOLMODEL.2023.110452

[CR64] Pesticide Properties DataBase (PPDB) (2024): http://sitem.herts.ac.uk/aeru/ppdb/en/ Accessed 14 May 2024

[CR66] R Core Team (2024) R: a language and environment for statistical computing. R Foundation for Statistical Computing, Vienna

[CR67] Rahimi E, Barghjelveh S, Dong P (2021a) Estimating landscape structure effects on pollination for management of agricultural landscapes. Ecol Process 10:59. 10.1186/S13717-021-00331-3/FIGURES/3

[CR68] Rahimi E, Barghjelveh S, Dong P et al (2021b) PollMap: a software for crop pollination mapping in agricultural landscapes. J Ecol Environ 45:255–263. 10.1186/S41610-021-00210-0/FIGURES/5

[CR69] Reemer M, Kleijn D (2012) Wilde bestuivers in appel: en perenboomgaarden in de Betuwe in 2010 en 2011

[CR70] Robinson AC, Peeler JL, Prestby T et al (2021) Beescape: characterizing user needs for environmental decision support in beekeeping. Ecol Inform 64:101366. 10.1016/J.ECOINF.2021.101366

[CR71] Rundlöf M, Stuligross C, Lindh A et al (2022) Flower plantings support wild bee reproduction and may also mitigate pesticide exposure effects. J Appl Ecol 59:2117–2127. 10.1111/1365-2664.14223

[CR72] Schmolke A, Abi-Akar F, Hinarejos S (2019) Honey bee colony-level exposure and effects in realistic landscapes: an application of BEEHAVE simulating clothianidin residues in corn pollen. Environ Toxicol Chem 38:423–435. 10.1002/ETC.4314 30575066 10.1002/etc.4314 PMC6850421

[CR73] Sciaini M, Fritsch M, Scherer C, Simpkins CE (2018) NLMR and landscapetools: an integrated environment for simulating and modifying neutral landscape models in R. Methods Ecol Evol 9:2240–2248. 10.1111/2041-210X.13076

[CR74] Senapathi D, Goddard MA, Kunin WE, Baldock KCR (2017) Landscape impacts on pollinator communities in temperate systems: evidence and knowledge gaps. Funct Ecol 31:26–37. 10.1111/1365-2435.12809/SUPPINFO

[CR75] Theodorou P, Radzevičiūtė R, Lentendu G et al (2020) Urban areas as hotspots for bees and pollination but not a panacea for all insects. Nat Commun 11:576. 10.1038/s41467-020-14496-6 31996690 10.1038/s41467-020-14496-6 PMC6989530

[CR76] Tisma A, Meijer J (2018) Lessons learned from spatial planning in the Netherlands. In support of integrated landscape initiatives, globally. Netherlands Environmental Assessment Agency. The Hague

[CR77] Van Den Brink PJ, Alix A, Thorbek P et al (2021) The use of ecological models to assess the effects of a plant protection product on ecosystem services provided by an orchard. Sci Total Environ 798:149329. 10.1016/j.scitotenv.2021.149329 34375230 10.1016/j.scitotenv.2021.149329

[CR78] Vasiliev D, Greenwood S (2020) Pollinator biodiversity and crop pollination in temperate ecosystems, implications for national pollinator conservation strategies: mini review. Sci Total Environ 744:140880. 10.1016/J.SCITOTENV.2020.140880 32693283 10.1016/j.scitotenv.2020.140880

[CR79] Zattara EE, Aizen MA (2021) Worldwide occurrence records suggest a global decline in bee species richness. One Earth 4:114–123. 10.1016/J.ONEEAR.2020.12.005

[CR80] Zenono (2023) CCNicholson/SEMLE: SEMLE_0.2 (0.2). 10.5281/zenodo.10066592

[CR81] Ziółkowska E, Topping CJ, Bednarska AJ, Laskowski R (2021) Supporting non-target arthropods in agroecosystems: modelling effects of insecticides and landscape structure on carabids in agricultural landscapes. Sci Total Environ 774:145746. 10.1016/J.SCITOTENV.2021.145746 33610978 10.1016/j.scitotenv.2021.145746

[CR82] Zurbuchen A, Landert L, Klaiber J et al (2010) Maximum foraging ranges in solitary bees: only few individuals have the capability to cover long foraging distances. Biol Conserv 143:669–676. 10.1016/J.BIOCON.2009.12.003

