# Supplementary material for: Understanding interactive effects between habitat configuration and pesticide use for pollination: towards better informed landscape management
Source: Ecol Process. 2025 Mar 3;14(1):25. doi: 10.1186/s13717-025-00587-z (PMC11876248; doi:10.1186/s13717-025-00587-z)
Supplement: Supplementary file 1 — Supplementary material 1. [file 13717_2025_587_MOESM1_ESM.docx]

Supplementary Material

We modelled pollination for several foraging ranges in a preliminary analysis. The results of this analysis revealed that the landscape dimensions, cell size, and foraging range interacted with the clustering of land uses. As visualized in Figure 1, the land use patterns within a short foraging range are almost identical to the ones within a larger foraging range in a landscape with a bigger cell size. Calculating pollination within multiple foraging ranges would therefore create similar results, but at slightly different positions along the gradient of land use clustering.


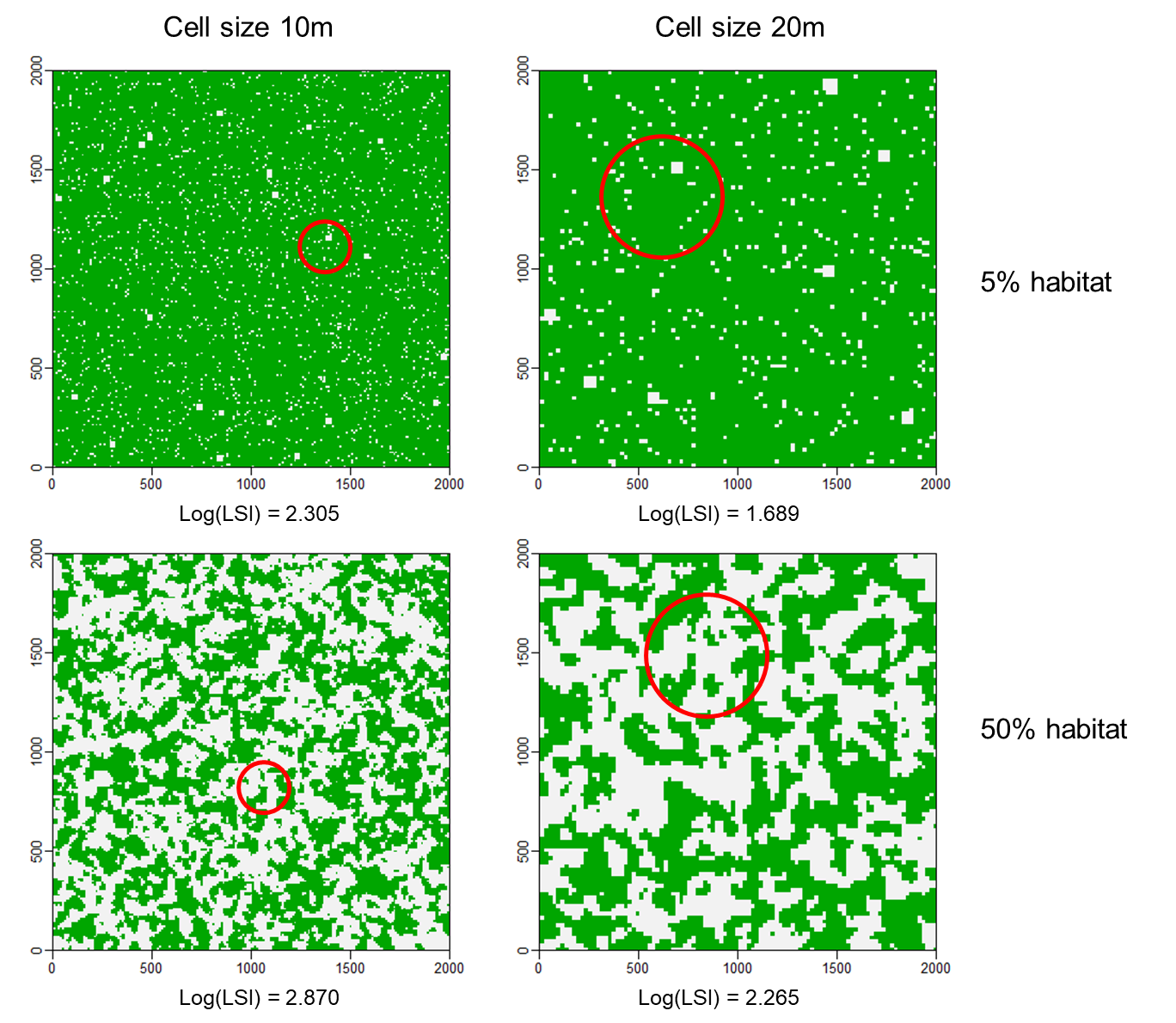


Figure 1: Comparison of land use patterns for landscapes differing in cell size, habitat percentage, and land use clustering (in log-transformed landscape shape index). Larger foraging ranges cover bigger parts of the landscape, but due to the bigger cell size the patterns that are “seen” by the pollinators are analogous to each other (red rings).
